# Whole-Genome Sequence of *Staphylococcus aureus* S54F9 Isolated from a Chronic Disseminated Porcine Lung Abscess and Used in Human Infection Models

**DOI:** 10.1128/genomeA.01207-15

**Published:** 2015-10-22

**Authors:** Bent Aalbæk, Louise Kruse Jensen, Henrik Elvang Jensen, John Elmerdahl Olsen, Henrik Christensen

**Affiliations:** Department of Veterinary Disease Biology, Faculty of Health and Medical Sciences, University of Copenhagen, Copenhagen, Denmark

## Abstract

We obtained a draft genome sequence of *Staphylococcus aureus* strain S54F9, which was isolated from a chronic disseminated porcine lung abscess and used in porcine infection models. Genes coding for a number of toxins, including enterotoxins and superantigen, were demonstrated in this strain.

## GENOME ANNOUNCEMENT

*Staphylococcus aureus* strain S54F9 was isolated in 2005 from a chronic embolic porcine lung abscess and has been applied in a number of porcine models of human infections, including hematogenous osteomyelitis (1–4), sepsis (5, 6), pyemia (7), endocarditis (8), and encephalitis (9). The strain was found to be useful in these types of porcine model infections, and information on relevant doses for inducing various degrees of sepsis has been obtained (5). In a comparative study, the strain was found to have a higher virulence in pigs than that of two human *S. aureus* strains, NCTC8325-4 and UAMS-1 (3).

The genome was sequenced with MiSeq (Illumina, San Diego, CA) using the paired-end method. The genome was assembled by CLC Genomics Workbench 7.0 (CLC bio, Qiagen, Hilden, Germany), with a quality trim level of 0.01, resulting in 3,688,424 reads with an average length of 248 nucleotides (nt), which assembled into 51 contigs >1,000 nt and resulted in a total genomic length of 2,775,659 nt. The G+C content was 32.8%. The genome has been annotated by the NCBI Prokaryotic Genome Automatic Annotation Pipeline (PGAAP).

Based on an analysis of the genome sequence, the strain was found to belong to *spa* type t1333 and multilocus sequence type (MLST) ST433, confirming the results of previous typing (10). Genes coding for a number of toxins, such as enterotoxins, including phage-associated enterotoxins, exotoxins, and superantigen, were demonstrated.

Most isolates of *S. aureus* from pigs have been found to cluster into three clonal complexes, namely, clonal complex 398 (CC398) (39%), CC30 (29%), and CC9 (27%) (4). A single *spa* type in each CC has been found to be predominant, namely, *spa* type t034 in ST398 of CC398, *spa* type t1333 in ST433 of CC30, and *spa* type t337 in ST9 of CC9 (10). The present strain S54F9 thus belongs to one of these three predominating porcine clonal complexes, having *spa* type t1333 and ST433 of CC30. The ability to form biofilms has been demonstrated in *S. aureus* strain S54F9 (3). Evidence points toward osteomyelitis being a biofilm-based infection (11). The developed porcine osteomyelitis model (3) is a tool for future studies of bacterial biofilms in hematogenous and implant-associated osteomyelitis *in vivo*. A well-characterized inoculation strain is essential for obtaining reproducible results in animal infection models of human disease. Strain S54F9 is useful for the development of porcine infection models in the future based on the detailed experiences gained with many types of porcine infection models.

### Nucleotide sequence accession number.

The whole-genome shotgun project has been deposited at DDBJ/EMBL/GenBank under the accession no. LIPH00000000. The first version is described here. The BioSample and BioProject numbers are SAMN04002937 and PRJNA293432, respectively.
